# Relationship Between Rheumatoid Arthritis and Periodontal Disease—Systematic Review and Meta-Analysis

**DOI:** 10.3390/jcm14010010

**Published:** 2024-12-24

**Authors:** Sabino Dolcezza, Javier Flores-Fraile, Ana Belén Lobo-Galindo, José María Montiel-Company, Álvaro Zubizarreta-Macho

**Affiliations:** 1 Faculty of Dentistry, Alfonso X El Sabio University, 28691 Madrid, Spain; 2 Department of Surgery, Faculty of Medicine and Dentistry, University of Salamanca, 37008 Salamanca, Spain; 3 Department of Stomatology, Faculty of Medicine and Dentistry, Universitat de Valéncia, 46010 Valencia, Spain; jose.maria.montiel@uv.es

**Keywords:** periodontitis, autoimmune disease, rheumatoid arthritis, periodontal disease, autoimmune

## Abstract

**Background/Objectives:** The aim of this systematic review and meta-analysis was to determine the association between rheumatoid arthritis and periodontal disease. **Methods:** This systematic review and meta-analysis of the scientific literature was carried out based on the recommendations of Preferred Reporting Items for Systematic Reviews and Meta-Analyses (PRISMA). We analyzed all studies that evaluated the relationship between the chronic inflammatory diseases through the response to non-surgical periodontal treatment, comparing the values of CAL (Clinical Attachment Level) for PD (periodontal disease) and DAS28 for RA. A total of three databases were searched in the literature search: Pubmed, Scopus, and Web of Science. After eliminating duplicate articles and applying certain inclusion criteria, of the 29 articles found, a total of 6 were included in the present study. **Results:** A statistically significant difference in mean reduction of −0.56 mm was obtained for CAL, with a 95% confidence interval of the difference between −0.82 and −0.31 (z-test = −4.33; *p*-value = 0.001) in favor of the periodontal treatment group. The heterogeneity of the meta-analysis was slight (I2 = 39% and Q = 8.19; *p*-value = 0.146). For DAS28, treatment showed a mean reduction of −0.39 DAS points, with a 95% CI between −0.46 and −0.31 (z-test = −10.3; *p*-value < 0.001) among patients with PD and RA. **Conclusions:** The present study shows how the control of periodontal disease through non-surgical periodontal treatment can reduce the severity of RA. This finding consistently supports the idea that there is a pathogenic association between these two chronic inflammatory diseases.

## 1. Introduction

The human microbiome encompasses all microorganisms inhabiting the skin, mucous membranes, and intestinal tract. The periodontium, a dynamic structure with specialized tissues, vasculature, lymphatics, and nerves, supports the teeth through components like alveolar bone, root cementum, periodontal ligament, and gingival mucosa [1,2,3,4,5,6,7,8,9,10,11,12,13,14]. Periodontal disease, a multifactorial inflammatory condition, progressively destroys these tissues, beginning with gingivitis-induced connective tissue degradation and mucosal disintegration [15,16,17,18,19,20,21,22,23,24,25,26,27,28,29,30].

Factors such as dental plaque and systemic conditions contribute to periodontal disease, which may lead to systemic complications like endocarditis, respiratory and coronary diseases, cerebrovascular events, diabetes control issues, pregnancy complications, and autoimmune diseases [31,32,33,34,35,36,37,38,39,40,41,42,43,44,45,46,47,48,49,50,51,52]. Autoimmune conditions like Sjögren’s syndrome, Crohn’s disease, systemic sclerosis, systemic lupus erythematosus, and rheumatoid arthritis (RA) have significant odontostomatological impacts. RA, a chronic autoimmune disease affecting 0.5–1% of adults globally, is mediated by inflammatory cascades involving immune complexes, leading to joint destruction [53,54,55,56].

RA predominantly affects women aged 40–50 and can impact various joints, including the TMJ, spine, and dentoalveolar joint. Chronic oral inflammation shares pathogenic mechanisms with RA, including leukocyte infiltration and cytokine release (e.g., TNF-α, ILs, GM-CSF, RANKL, and MMPs) [57,58,59]. Periodontal bacteria are implicated in autoimmune pathology and found in high levels in RA patients’ serum and synovial fluid, underscoring a pathogenic link between periodontitis and RA-associated osteoarticular degeneration [60,61].

The aim of this work was to determine the association between rheumatoid arthritis and periodontal disease, with a null hypothesis (H_0_) stating that there is no association between rheumatoid arthritis and periodontal disease.

## 2. Materials and Methods

### Study Design

The literature review was conducted following the guidelines for systematic reviews and meta-analyses in accordance with PRISMA (Preferred Reporting Items for Systemic Reviews and Meta-Analyses: http://www.prisma-statement.org (accessed on 4 May 2020); International Prospective Register of Systematic Reviews (PROSPERO) registration number: CRD42020192179). The review also complied with the PRISMA 2009 Checklist [62] and was performed in accordance with current recommendations regarding systematic reviews and meta-analyses. The population, intervention, comparison, and outcome (PICO) question was “Is there a link between periodontal disease and rheumatoid arthritis?” The following components were considered: Population: patients affected with periodontal disease and affected by rheumatoid arthritis; Intervention: non-surgical periodontal treatment; Comparison: patients submitted and non-submitted to periodontal treatment; Outcome: clinical attachment loss (CAL) and DAS28.

An electronic search was carried out using the following databases: PubMed, Scopus, Cochrane, and Web of Science. The search assessed all the literature published internationally up to March 2024. Five medical subject heading (MeSH) terms were included in the search: “Periodontitis”; “autoimmune disease”; “rheumatoid arthritis”; “periodontal disease”; “randomized clinical trial”. One Boolean operator was applied (“AND”). These search terms were applied as follows: [(periodontitis) AND (autoimmune disease) AND (rheumatoid arthritis) AND (randomized clinical trial)]. Two different researchers (S.D.; A.Z.M.) searched the databases simultaneously. The inclusion and exclusion criteria were applied to titles, and a single researcher (S.D.) extracted the data regarding the relevant variables. A.Z.M. conducted the systematic review, and two researchers who had not participated in the selection process (A.Z.M.; S.D.) performed the subsequent meta-analysis.

The inclusion criteria were as follows: randomized clinical trials; samples of patients of any age affected by periodontitis or periodontal diseases and rheumatoid arthritis. Studies were not restricted by language or year of publication. The exclusion criteria were as follows: systematic reviews of the literature, clinical cases, case series, and editorials; studies that included patients affected by another oral disease, by another autoimmune disease, or whose periodontitis or rheumatoid arthritis was related to another type of pathology. The following data were recorded: author, year, title, journal, and sample size (*n*).

The risk of bias of the clinical studies selected for review was assessed using the Jadad scale for the methodological quality assessment of clinical trials. The Jadad scale consists of five items that evaluate randomization, researcher and patient blinding, and descriptions of losses during follow-up, producing a score of 0–5: scores of less than 3 are considered low quality [63].

The meta-analysis was carried out using the random effects model inverse variance method. The significance of the effect size, measured as the difference in means between the periodontal treatment group and the control group for the variables CAL (mm) and DAS28 score, was assessed with the z test. The heterogeneity of the meta-analysis was analyzed using the Q test and I^2^. The level of significance was established at *p* < 0.05. The meta-analysis is represented in a forest plot.

Publication bias was analyzed using the trim-and-fill method of funnel plot skew adjustment.

## 3. Results

### 3.1. Flow Diagram

**Figure 1:**
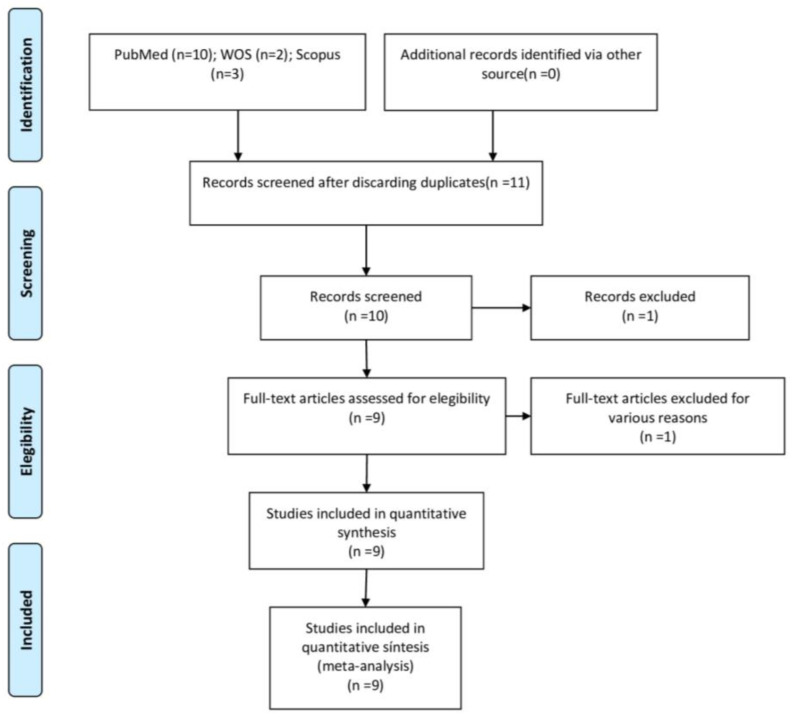
PRISMA flow diagram showing the preferred reporting items for systematic reviews and meta-analyses approach. The diagram illustrates the process of selecting studies for inclusion, starting from the identification of records through database searches, followed by screening for eligibility, and concludes with the final number of studies included in the review. The flowchart highlights the various stages of the review process, including exclusions and reasons for exclusion at each stage.

Applying the selection criteria pre-established in the search strategy, a total of 29 articles were obtained: 21 from PubMed, 5 from Web of Science, and 3 from Scopus. Of the total of 29 articles, 3 were discarded as duplicates. After reading the titles, abstracts and having screened the articles according to the objective of this meta-analysis, another 20 were eliminated, leaving a total of 6. The six articles were ultimately assessed in the quantitative analysis, as they included all the necessary data and variables, and met all the selection criteria (Figure 1).

### 3.2. Qualitative Analysis

Table 1 shows the qualitative analysis. Of the 21 articles found, all 6 selected were randomized clinical trials [4,6,12,13,14,15,16,17]. Most of the values used are expressed as mean and standard deviation, except for one measured in IQR [1]. The measurements of CAL, expressed in mm, and DAS28, in standard units, were compared to demonstrate the relationship between the two pathologies. Sample sizes were below 100 patients, and no dropouts or rejections of experimental studies were detected. In all the studies, a control group and an experimental group were detected to serve as a comparison when demonstrating the relationship between the diseases using non-surgical periodontal treatment and medical laboratory analyses as means of measurement.

### 3.3. Quality Assessment

Table 2 shows the results of the methodological quality assessment using the JADAD scale. In most of the studies, there was a description of losses and retreats. It should also be noted that not all the articles were described as double-blind, except in the case of Silvestre-Rangil et al. [60] and Thilagar et al. [61], although most were detected as randomized.

### 3.4. Quantitative Analysis

#### 3.4.1. CAL

**Figure 2:**
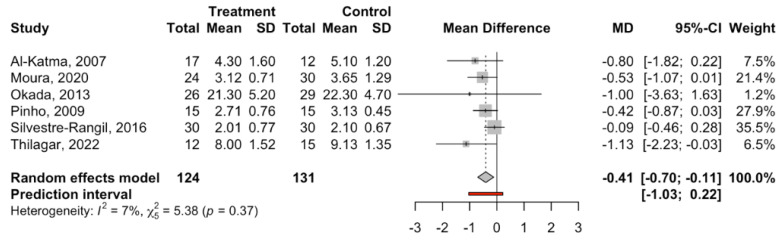
Forest plot of the meta-analysis of the difference in means (CAL in mm) [53,55,57,58,60,61].

The six studies were combined using the random effects model inverse variance method to estimate the effect size via the mean difference in CAL (mm) between the periodontal treatment (*n* = 124) and control groups (*n* = 131), obtaining a statistically significant difference of −0.41 mm with a 95% confidence interval between −0.70 and −0.12 (z test = −2.73; *p*-value = 0.006) in favor of the periodontal treatment group. The meta-analysis did not present heterogeneity, with an I2 = 7% and a Q test = 5.38; *p*-value = 0.372 (Figure 2).

#### 3.4.2. Publication Bias

**Figure 3:**
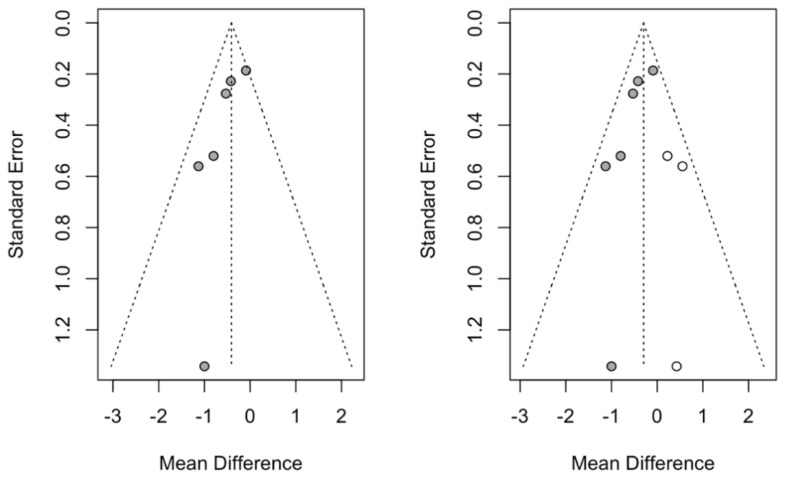
Funnel plots of the meta-analysis of the mean difference in CAL in mm with the trim-and-fill method, showing the initial estimate (**left funnel plot**) and the estimate with the 3 studies added as white points (**right funnel plot**).

Publication bias was studied using the trim-and-fill method of adjusting the asymmetry of the funnel plot; by adding three studies, the effect size was estimated at −0.30 mm, with a 95% CI between–0.55 and −0.046 (z test = −2.31: *p*-value = 0.021). The estimate, with the addition of the studies, maintains a significant reduction in mm in favor of the treatment group over the control, which confirms that the analysis is free of publication bias (Figure 3).

#### 3.4.3. DAS28

**Figure 4:**
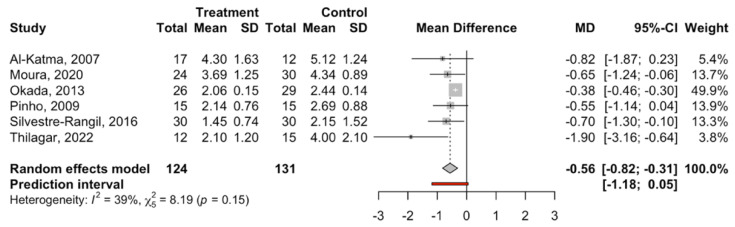
Forest plot of the meta-analysis of mean difference (DAS28 score) [53,55,57,58,60,61].

Six studies were combined using the random effects model inverse variance method to estimate the effect size via the difference in means of the DAS28 score between the periodontal treatment and control groups, obtaining a statistically significant difference of −0.56 with a 95% confidence interval of the difference between −0.82 and −0.31 (z test = −4.33; *p*-value = 0.001) in favor of the periodontal treatment group. The heterogeneity of the meta-analysis was slight (I2 = 39% and Q test = 8.19; *p*-value = 0.146) (Figure 4).

#### 3.4.4. Publication Bias

**Figure 5:**
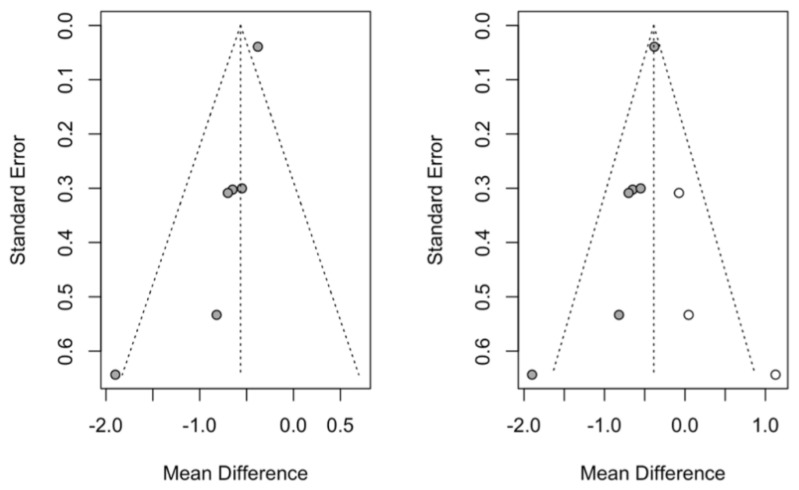
Funnel plots of the meta-analysis of the mean difference in DAS28 score with the trim-and-fill method, showing the initial estimate (**left funnel plot**) and the estimate with the 3 studies added as white points (**right funnel plot**).

In adjusting the asymmetry of the funnel plot, three studies were added, so the effect size was re-estimated as −0.39 DAS28 points, with a 95% CI between −0.46 and −0.31 (z test = −10.3; *p*-value < 0.001). The new estimate, with the addition of the studies, maintains a significant reduction in DAS28 score in favor of the periodontal treatment group compared to the control, so again, we can affirm that the analysis is not affected by publication bias (Figure 5).

## 4. Discussion

The results of the present study support the null hypothesis (H0), which posits that no significant association exists between rheumatoid arthritis (RA) and periodontal disease. However, our findings demonstrate the beneficial effects of non-surgical periodontal treatment in alleviating RA symptoms, as evidenced by the significant reduction in DAS-28 scores observed in the experimental groups of the included studies. Additionally, non-surgical treatment resulted in a decrease in periodontal pathogen levels and significantly improved or stabilized Clinical Attachment Level (CAL) parameters in individuals with both periodontal disease (PE) and RA.

It is noteworthy that while the control groups exhibited a significant worsening or progression of both inflammatory conditions, the treatment groups experienced a stabilization of these values over the 2-month study period. This stabilization in the treatment groups was likely aided by weekly periodontal exams, prophylaxis, and the reinforcement of oral hygiene instructions. CAL scores, which are based on a threshold of 4 mm—indicating active periodontal disease—showed an increase in the control groups, reflecting the progression of periodontitis. Conversely, in the treatment groups, a statistically significant improvement in CAL was observed, with a mean difference of −0.56 and a 95% confidence interval ranging from −0.82 to −0.31 (z-test = −4.33; *p*-value = 0.001). These same improvements were observed concurrently with improvements in RA indices.

Furthermore, a strong correlation (r = 0.97) was found between DAS28 and the original DAS (inflamed joint count). DAS28, which has a continuous scale ranging from 0 to 9.4, categorizes disease activity as low (DAS28 ≤ 3.2), moderate (3.2 < DAS28 ≤ 5.1), or high (DAS28 > 5.1). The patients selected for the study initially showed moderate RA activity. However, in the treatment groups, reductions in DAS28 scores were observed, shifting from moderate-to-low disease activity, in contrast to the control groups, which showed worsening scores. The mean reduction in DAS28 was −0.39 points, with a 95% confidence interval between −0.46 and −0.31 (z-test = −10.3; *p*-value < 0.001).

These results highlight the positive and favorable effects of non-surgical periodontal treatment at both the microbiological and biological levels in periodontal and joint tissues. In summary, the findings suggest that controlling periodontal infections and gingival inflammation through scaling/root planing and plaque control in individuals with moderate periodontal disease may contribute to a reduction in the signs and symptoms of active rheumatoid arthritis.

## 5. Conclusions

The conclusions of this study suggest that controlling periodontal disease through non-surgical periodontal treatment may help reduce the severity of rheumatoid arthritis (RA). This finding supports the potential association between these two chronic inflammatory conditions. Given this, a multidisciplinary approach is recommended for patients with RA, emphasizing the importance of diagnosing any periodontal involvement and incorporating non-surgical periodontal therapy alongside conventional antirheumatic treatments. However, further research is needed to fully understand the extent of this relationship and its clinical implications.

## Figures and Tables

**Table 1:** Qualitative analysis of articles forming part of the systematic review.

Author (Year)	Study Type	Sample (*n*)	Follow-Up Time (Months)	Measurement Procedure	CAL (mm)	DAS28	*p* Value
Al-Katma et al. (2007) [53]	RCT	28	2	Clinical and radiographic	1.7 ± 0.8 (Contr.)	4.6 ± 1.1 (Contr.)	*p* < 0.05
					1.4 ± 0.6 (Treat.)	4.3 ± 1.6 (Treat.)	
Moura et al. (2020) [55]	RCT	30	2	Clinical and radiographic	3.5 ± 1.8 (Contr.)	4.34 ± 0.89 (Contr.)	*p* < 0.001
					2.4 ± 1.3 (Treat.)	3.69 ± 1.25 (Treat.)	
Nguyen et al. (2021) [56]	RCT	38	6	Clinical and radiographic	IQR 1.5–2.8 (Contr.)	IQR 3.3–4.5 (Contr.)	*p* < 0.001
					IQR 1.1–1.8 (Treat.)	IQR 2.5–4.0 (Treat.)	
Okada et al. (2013) [57]	RCT	55	2	Clinical and radiographic	3.10 ± 0.15 (Control)	2.44 ± 0.14 (Control)	*p* < 0.05
					2.85 ± 0.14 (Treat.)	2.06 ± 0.15 (Treat.)	
Pinho et al. (2009) [58]	RCT	75	6	Clinical and radiographic	3.12 ± 0.14 (Control)	2.69 ± 0.88 (Conrol)	*p* < 0.0001
					2.82 ± 0.77 (Treat.)	2.16 ± 0.76 (Treat.)	
Silvestre-Rangil et al. (2016) [60]	RCT	73	2	Clinical and radiographic	2.45 ± 0.82 (Control)	2.15 ± 1.54 (Control)	*p* < 0.0001
					2.17 ± 0.67 (Treat.)	1.45 ± 0.76 (Treat.)	
Thilagar et al. (2022) [61]	RCT	28	2–3	Clinical and radiographic	2.13 ± 1.52 (Control)	4.00 ± 0.40 (Control)	*p* < 0.001
					1.00 ± 1.35 (Treat.)	2.36 ± 1.20 (Treat.)	

RCT: Randomized clinical trial.

**Table 2:** Methodological quality assessment of clinical trials using the JADAD scale.

Author (Year)	Was the Study Described as Randomized?	Is the Method for Generating the Randomization Sequence Described, and Is This Method Appropriate?	Is the Study Described as Double-Blind?	Is the Blinding Method Described, and Is This Method Appropriate?	Was There a Description of Losses and Withdrawals?
Al-Katma et al. (2007) [53]	Yes	Yes	No	No	Yes
Moura et al. (2020 [55]	Yes	No	No	No	Yes
Okada et al. (2013) [57]	Yes	Yes	No	No	Yes
Pinho et al. (2009) [58]	Yes	Yes	No	No	Yes
Silvestre-Rangil et al. (2016) [60]	Yes	Yes	Yes	Yes	No
Thilagar et al. (2022) [61]	Yes	Yes	Yes	Yes	Yes

## Data Availability

Information is available upon request in accordance with relevant restrictions (e.g., privacy or ethical).
